# The Co-Delivery of Natural Products and Small RNAs for Cancer Therapy: A Review

**DOI:** 10.3390/molecules30071495

**Published:** 2025-03-27

**Authors:** Xuyi Wang, Shuang Li, Zelong Wang, Baorong Kang, Hong Yan

**Affiliations:** School of Pharmacy, Hunan University of Chinese Medicine, Changsha 410208, China; 20223748@stu.hnucm.edu.cn (X.W.); 20233789@stu.hnucm.edu.cn (S.L.); 20243809@stu.hnucm.edu.cn (Z.W.); 20243788@stu.hnucm.edu.cn (B.K.)

**Keywords:** nano-DDS, cancer, gene, natural products, co-delivery, RNA

## Abstract

This review summarizes the research progress in the co-delivery of natural products (NPs) and small RNAs in cancer therapy. NPs such as paclitaxel, camptothecin, and curcumin possess multi-target antitumor effects, but their applications are limited by drug resistance and non-specific distribution. Small RNAs can achieve precise antitumor effects through gene regulation, yet their delivery efficiency is low, and they are prone to degradation by nucleases. Nanomaterial-based drug delivery systems (nano-DDSs) provide an efficient platform for the co-delivery of both, which can enhance the targeting of their delivery and improve the synergistic antitumor effects simultaneously. The mechanisms of the antitumor action of natural compounds and small RNAs, the design and application of nanocarriers, and the latest research progress in co-delivery systems are introduced in detail in this paper. The application prospects of the co-delivery of natural compounds and small RNAs in cancer therapy are also discussed.

## 1. Introduction

Currently, cancer is still defined as a major public health issue worldwide and is the second leading cause of death globally [1]. In traditional cancer treatment, surgery, chemotherapy, and radiotherapy are the main methods for treating localized tumors [2]. Chemotherapy drugs, being non-targeted, can cause toxicity to normal organs and tissues due to their systemic effects [3]. Radiation therapy can directly damage the DNA of tumor cells, killing them or inhibiting their growth. However, it can also harm surrounding normal tissues, causing local side effects [4]. Moreover, during long-term drug therapy, tumor cells can easily develop drug resistance, especially multidrug resistance (MDR) [5]. The development of MDR is one of the main reasons for the failure of cancer treatment, which can lead to rapid cancer recurrence or disease progression and ultimately cause patient death [6]. Therefore, there is an urgent need to develop new therapeutic approaches to overcome the limitations of conventional treatments.

**Figure 1:**
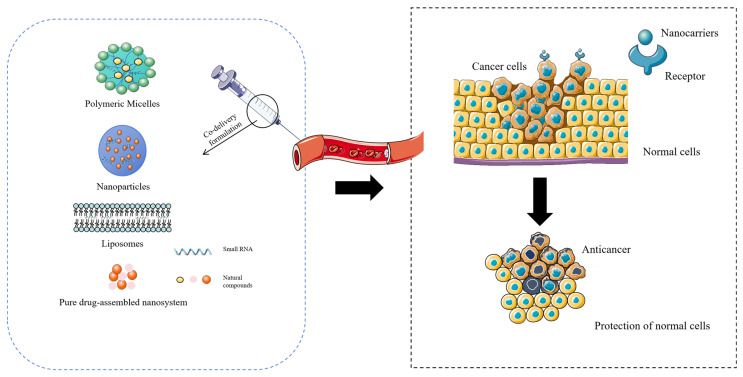
Schematic diagram of antitumor effects of co-delivery formulations of NPs and small RNAs.

NPs typically have multi-target antitumor effects and can promote combined antitumor actions in multiple ways [7], such as by inducing tumor cell apoptosis, inhibiting tumor angiogenesis, inducing tumor cell autophagy, and modulating the tumor microenvironment [8]. Also, NPs can boost the body’s immune response to tumors and improve treatment results [9]. NPs are very important in the research on new antitumor drugs. Many molecular-targeted antitumor drugs that come from NPs or their derivatives are now used for cancer treatment or are in clinical trials [10]. Tumor cells often have MDR to antitumor ingredients; thus, single NPs are not ideal for antitumor treatment [11]. Moreover, long-term treatment may also cause toxic side effects, which further limit their use in the clinic [12]. So, it is important to choose a co-delivery “partner” that can help NPs work better against tumors. In the human genome’s RNA, only 3% is transcribed into coding mRNA, while the non-protein-coding RNA is called ncRNA [13]. We have noticed that non-coding RNA (ncRNA), which regulates gene expression, has become a key player in gene regulation [14]. Depending on their length and location, ncRNAs are further classified into lncRNAs and small ncRNAs [15]. Small ncRNAs, also known as small RNAs, are an important member of the ncRNA family and have profoundly influenced the field of RNA biology and other areas [16]. Small RNAs refer to RNA molecules that are 18 to 30 nucleotides in length. They play important roles in gene expression regulation and many biological processes [17]. They mainly work by specifically inhibiting or upregulating the expression of certain genes through RNA interference (RNAi) and RNA activation (RNAa) techniques. Since the transformation of normal cells into malignant cells is associated with the dysregulation of gene expression [18], small RNAs, as a precise, specific, and highly effective gene regulation therapy, have attracted much attention in the field of tumor-targeted therapy. They inhibit the growth and metastasis of tumors by precisely targeting genes related to the proliferation, migration, invasion, and apoptosis of tumor cells. However, the current challenge in treatment is that neither single NPs nor single small RNAs alone can achieve satisfactory results in treating tumors. Therefore, the combined use of NPs and small RNAs has become a significant new strategy to improve the effectiveness of cancer treatment and increase patient survival rates. The delivery efficiency of both NPs and small RNAs greatly affects their therapeutic efficacy. NPs usually distribute nonspecifically in the body. Long-term administration may cause toxicity to normal cells, and many natural compounds have poor water solubility [19]; these reasons have affected the bioavailability of the drugs [20]. As nucleic acid molecules, small RNAs are easily degraded by nucleases, and their off-target effects can also impact normal cells [21]. Nanoparticulate drug delivery systems (nano-DDSs) provide an excellent carrier platform for the efficient delivery of antitumor drugs [22]. Nano-DDSs can enhance the targeting of drug delivery to tumors [23], improve the pharmacokinetic parameters of drugs in the body [24], and boost the synergistic antitumor effects of natural antitumor active ingredients and small RNAs, effectively overcoming multidrug resistance [25]. Nano-DDSs usually refer to nanoscale dispersion systems formed from natural or synthetic polymers and inorganic nanomaterials in various forms [26]. In addition to classical nanocarriers, self-assembled NPs with anticancer activity have provided many new ideas for novel drug delivery systems in the biomedical field [27]. Since nano-DDSs have the potential to encapsulate both hydrophobic and hydrophilic drugs at the same time, they are well suited to the development of the co-delivery of targeted antitumor formulations [28]. Figure 1 expresses a nano-DDS entering the body through intravenous injection to exert antitumor effects. Various functional nanocarriers can promote cancer treatment by delivering one or more therapeutic agents to cancer tissues and cells with minimal off-target effects and appropriate release kinetics and dosages. Nanocarriers usually have passive targeting to tumors due to the enhanced permeability and retention (EPR) effect, and appropriate ligand modification during the construction of nanocarriers can enhance their active targeting. The figure illustrates examples of nanomicelles, nanoparticles, liposomes, and pure drug self-assembly as co-delivery systems. These systems, when administered via intravenous injection, can accumulate drugs in tumor tissues through the enhanced permeability and EPR effect or by the binding of ligands to receptors. The co-delivery design of NPs and small RNAs is gradually becoming a hot area in the research on targeted antitumor formulations. However, there is currently a lack of comprehensive reviews summarizing the progress in this field, and exploration in this area may provide key parameters for the synergistic antitumor effects of drugs. In this review, we summarize the research achievements regarding nano-DDSs in co-delivery for cancer treatment in recent years, systematically review the synergistic mechanisms of different natural compounds and small RNAs, and focus on the latest research progress in and potential application prospects of co-delivery nanoformulations of NPs and small RNAs.

## 2. Antitumor Effects of Natural Products

### 2.1. Paclitaxel (PTX)

Many natural compounds and their derivatives have been applied in clinical practice or are undergoing clinical trials [29]. PTX is a natural alkaloid isolated from the bark of *Taxus brevifolia* [30] and, as the first approved natural compound, has been widely used in the clinical treatment of various cancers, including ovarian, breast, and lung cancers [31]. The core mechanism of PTX is to bind to tubulin and stabilize the microtubule structure, thereby preventing normal cell division and arresting cells in the G2/M phase, thereby inhibiting the proliferation of tumor cells [32]. Because the tumor cell cannot complete mitosis normally, cell cycle arrest will lead to a series of stress responses within the cell, eventually activating the cell apoptosis pathway [33]. PTX can also inhibit the proliferation and migration of tumor vascular endothelial cells, disrupt the formation of new tumor blood vessels, block the blood supply to tumor tissues, and thereby improve the tumor microenvironment [34]. However, the poor water solubility and limited availability of PTX also restrict its application [35]. To address these issues, researchers are actively developing PTX derivatives with the same mechanism of action, such as docetaxel [36]. Researchers are also exploring more precise delivery carriers to improve the antitumor effects of poorly water-soluble PTX. Several formulations of PTX and its derivatives have been approved for marketing [37]. Albumin-bound paclitaxel can effectively enhance the antitumor effects of PTX. For example, Abraxane^®^, an albumin-bound paclitaxel formulation, was approved by the FDA in 2005 for the first-line treatment of locally advanced or metastatic non-small-cell lung cancer [38]. PTX must be dissolved in a lipid solvent. However, using solvents such as polyoxyethylated castor oil and dehydrated ethanol can increase the side effects of PTX [39]. Encapsulating PTX in liposomes for delivery can effectively address the issue of its poor water solubility [40]. Lipusu^®^, the first approved paclitaxel liposome, is a first-line chemotherapeutic agent for ovarian cancer and its metastatic treatment [41]. It is the first paclitaxel liposome approved worldwide [42]. In addition, many studies have demonstrated that the combination of PTX and gene therapy can reverse the MDR of tumor cells to PTX [43]; this combined therapeutic strategy enhances the antitumor effects of PTX and has become one of the current research hotspots in PTX antitumor studies.

### 2.2. Camptothecin (CPT)

CPT is a cytotoxic quinoline alkaloid isolated from the bark and branches of *Camptotheca acuminata*, *Nyssaceae* (also known as the happiness tree), which is unique to China. It has unique anticancer activity and is commonly used in the treatment of colorectal cancer, ovarian cancer, and gastric cancer [44]. The primary antitumor mechanism of CPT is through the inhibition of topoisomerase I (Topo I) [45]. Topo I is a key enzyme that relieves the superhelical tension of DNA during DNA replication, allowing DNA to unwind smoothly [46]. CPT can bind to the Topo I-DNA complex, stabilizing this complex and preventing the reconnection of the DNA strands, leading to DNA damage [47]; this mechanism is particularly lethal to rapidly proliferating tumor cells. Due to the induction of DNA damage and apoptosis, CPT can effectively block the cell cycle, especially in the S phase. This blocking effect inhibits the proliferation and division of tumor cells. CPT can also further inhibit the growth of cancer cells by interfering with the transcription process of RNA polymerase. Poor water solubility and susceptibility to hydrolysis in aqueous media are important factors that limit the effectiveness of CPT [48]. Additionally, CPT is toxic, and its toxic effects on normal cells under physiological pH should not be overlooked [49]. CPT is also susceptible to enzymes such as cytochrome P450 (CYP450), leading to increased drug interactions and toxicity [50]. Irinotecan and topotecan, derivatives of CPT, are also effective anticancer drugs, and they were approved by the FDA in 1996 [51]. Delivering CPT and its derivatives using nanocarriers can effectively reduce their side effects. Onivyde^®^ is an irinotecan liposome, used clinically as a Topo I inhibitor to treat pancreatic cancer [52].

### 2.3. Curcumin (Cur)

Cur is a natural phenolic antioxidant extracted from the rhizome of the plant *Curcuma longa* [53]. Its molecular structure is unique, with the main chain consisting of unsaturated aliphatic and aromatic groups, forming a diarylheptanoid shape, and possessing the characteristics of both diketone compounds and the rare diketone structural pigments in the plant kingdom [54]. Cur exhibits broad-spectrum antitumor activity. Its antitumor effects are mainly achieved by inhibiting the activation of NF-κB, downregulating anti-apoptotic proteins, upregulating pro-apoptotic proteins, and inhibiting signaling pathways such as PI3K/Akt, thereby inducing cancer cell apoptosis and inhibiting tumor growth [55]. It is worth mentioning that Cur also has unique and potent antioxidant and anti-inflammatory effects. It can regulate various signaling pathways to inhibit the expression of inflammatory factors such as TNF-α and IL-6, thereby reducing the promoting effects of inflammation on tumors. [56]. However, the low bioavailability of Cur necessitates the selection of appropriate nanocarriers for its delivery [57]. Lipocurc™ is a liposome-encapsulated Cur formulation currently in the clinical trial stage. Experimental studies on Lipocurc™ have shown that liposome-encapsulated delivery can effectively increase the bioavailability of Cur [58]. Although no Cur nanoformulations have been officially approved for marketing yet, with the continuous development of nanotechnology and the advancement of clinical trials, it is expected that more efficient Cur nanoformulations will enter the market in the future.

### 2.4. Resveratrol (RES)

RES is a non-flavonoid polyphenolic organic compound widely found in foods such as grapes and red wine [59]. This NP is an antitoxin produced by many plants in response to environmental stress and has significant biological activity [60]. RES has shown good application prospects in the treatment of various cancers, including colorectal cancer, prostate cancer, and breast cancer [61]. RES exerts its anticancer effects through various mechanisms, including inhibiting tumor cell proliferation, inducing apoptosis, and suppressing inflammatory responses [62]. However, high doses of RES may cause side effects, such as gastrointestinal discomfort, and RES is rapidly metabolized in the body, and its metabolites have low antitumor activity [63]. Various resveratrol derivatives have been developed, such as pterostilbene, which have better bioavailability and anticancer activity [64]. Delivering RES via liposomes or solid lipid nanoparticles is also a common method to enhance its antitumor activity [65]. Currently, nanoformulations of RES have not been officially approved as clinical drugs.

## 3. Small-RNA Antitumor Effect Through Gene Regulation

**Figure 2:**
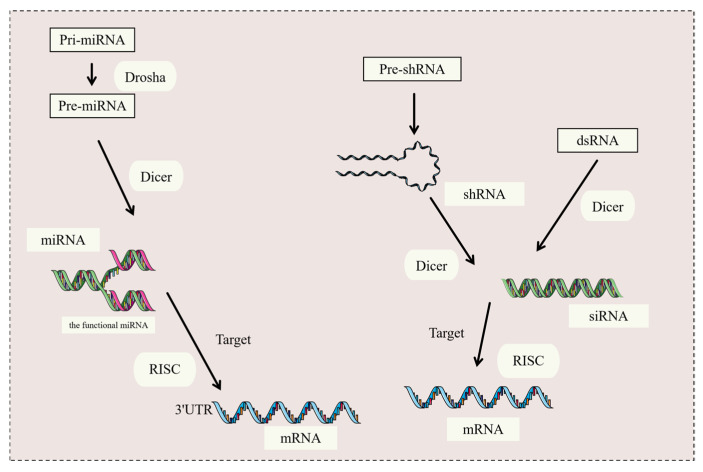
Generation processes of three kinds of small RNAs.

Small interfering RNA (siRNA) and microRNA (miRNA) are two important types of small RNAs that play significant roles in antitumor activity [66]. Based on a review of the literature, short hairpin RNA (shRNA) is also classified as a kind of small RNA and has been studied for co-delivery with NPs. Based on this, this paper will focus on the above three kinds of small RNAs [67]. Figure 2 illustrates the biogenesis processes of the three types of small RNAs mentioned above. Small RNAs can efficiently and specifically block the expression of specific genes in the body through RNA interference (RNAi). siRNA and shRNA induce mRNA degradation, thereby inhibiting gene expression. Moreover, miRNA binds to the 3′UTR of mRNA, leading to translational repression [68]. Because RNAi can specifically reduce the expression of target genes, thereby inducing gene silencing and regulating biological processes such as cell growth, development, gene transcription, and translation, it has been widely used in the field of cancer treatment [69]. Small RNAs are commonly designed to target and silence specific genes for cancer treatment [70].

### 3.1. The Biological Target of Small RNAs

Small RNAs have shown great potential in antitumor treatment by targeting various key molecules and pathways. Small RNAs can inhibit tumor growth by binding to the mRNAs of growth factor receptors such as EGFR, HER2, and VEGFR. For example, VEGF siRNA blocks tumor angiogenesis by inhibiting VEGF expression by RNAi [71]. Small RNAs can also genetically regulate cell cycle regulators such as Cyclin D1, Cyclin E, CDK 4, and CDK 6. Cyclin D1 is a key regulator of the cell cycle, capable of forming complexes with CDK4 and CDK6 to phosphorylate and activate cell cycle inhibitory proteins, thereby promoting the transition of the cell cycle from the G1 phase to the S phase [72]. miR-15a and miR-16 were able to regulate Cyclin D1 to promote its degradation and inhibit tumor cell proliferation [73]. Small RNAs can regulate apoptosis-inhibiting genes, such as Bcl-2, Bcl-xL, and Survivin. Survivin is a special member of the inhibitor of apoptosis protein (IAP) family, which is highly expressed in various tumor tissues but has low expression in normal mature tissues [74]. Researchers found that Survivin shRNA can specifically bind to Survivin mRNA and, in combination with miR-494, can synergistically silence the expression of the Survivin protein to induce apoptosis in tumor cells [75]. Small RNAs can also regulate the tumor-promoting transcription factors, such as c-Myc, NF-κB, and HIF-1α. c-Myc is a key transcription factor that is widely involved in the regulation of cell proliferation, differentiation, and apoptosis [76]. Researchers have used c-Myc siRNA to target c-Myc mRNA and have found that siRNA can significantly reduce the expression levels of c-Myc, thereby inhibiting the proliferation and survival of tumor cells [77]. In addition, small RNAs can also inhibit the negative regulators of genes, such as ZEB 1 and ZEB 2, which can restore the function of tumor suppressor genes and inhibit tumor cell invasion and metastasis [78]. By regulating the key molecules in the PI3K/Akt/mTOR pathway, Ras/Raf/MEK/ERK pathway, and Wnt/β-catenin pathway, small RNAs can inhibit the activation of the pathways and induce tumor cell apoptosis [79]. Research has found that delivering small RNAs using an effective delivery carrier can improve the tumor mircroenvironment (TME) and enhance the antitumor immune response [80]. Because of these multi-level mechanisms of action, small RNAs offer new strategies and ideas for antitumor treatment and have become a focal point in antitumor drug research.

### 3.2. Classification of Small RNAs

#### 3.2.1. Small Interfering RNA (siRNA)

Dicer is an endoribonuclease that is responsible for cleaving long double-stranded RNA (dsRNA) into siRNA, enabling it to be assembled into the RNA-induced silencing complex (RISC) [81]. siRNA is a type of small RNA composed of a sense strand and an antisense strand, typically 21–23 nucleotides in length, which is capable of inducing the silencing of complementary target mRNA [82]. After siRNA enters the cell, it is integrated into the RISC, and then the double-stranded structure of siRNA is unwound to form single-stranded siRNA [83]. Once the single-stranded siRNA successfully binds to the target mRNA, it induces the cleavage of the mRNA. The cleaved mRNA is recognized as an aberrant molecule by the cell and is subsequently degraded [84]. Because the mRNA cannot be translated into an amino acid sequence, the corresponding protein cannot be synthesized, thereby silencing the gene that encodes this mRNA and exerting an RNAi effect, which has become one of the key therapies for antitumor treatment [85]. In the field of tumor-targeted therapy, siRNA has attracted much attention and is expected to provide new strategies and means for cancer therapy.

#### 3.2.2. MicroRNAs (miRNAs)

miRNAs are a class of endogenous small-molecule ncRNAs, about 21 to 25 nucleotides in length, which are widely found in eukaryotes [86], and they specifically bind to the target mRNA, leading to the inhibition of the translation or degradation of the target mRNA, thereby regulating the translation process of proteins [87]. Unlike siRNA, which is double-stranded RNA, miRNA is single-stranded RNA. miRNA is initially present in the longer pri-miRNA form, and these transcripts often contain multiple hairpin structures [88]. The pri-miRNA is cleaved by the Drosha enzyme in the nucleus to form a shorter pre-miRNA. After the pre-miRNA is transported into the cytoplasm, it is further cleaved by the Dicer enzyme to produce mature miRNA duplexes [89]. Subsequently, the miRNA duplexes dissociate and the functional chain is incorporated into the RNA-induced RISC to play a regulatory role [90]. The miRNA regulates the mRNA stability and translation efficiency mainly through complementary binding to the 3′UTR of the mRNA [91]. After binding, miRNA can promote the degradation of mRNA or inhibit its translation, thereby reducing the amount of protein synthesis and achieving the negative regulation of gene expression [92]. A single miRNA can target multiple mRNAs, and a single mRNA can be regulated by multiple miRNAs. This many-to-many regulatory relationship allows miRNAs to finely regulate gene expression within cells [93].

#### 3.2.3. Short Hairpin RNA (shRNA)

shRNA, as the name suggests, is shaped similar to a short hairpin. shRNA is an artificially designed RNA molecule composed of both the sense and antisense strands [94]. The sense strand of shRNA is complementary to the target mRNA, and the antisense strand is connected to the sense strand through a loop structure, forming a short hairpin structure [95]. The sense and antisense strands of shRNA are usually about 21–23 nucleotides long each, and the circular structure is about 4–10 nucleotides in length [96]. The RNAi mechanism of shRNA is similar to that of siRNA. It is recognized and utilized by the RNA-induced RISC within the cell, guiding the RISC to bind to the target mRNA, leading to the degradation of the mRNA or the inhibition of its translation, thereby achieving gene silencing [97]. Unlike siRNA, which is chemically synthesized, double-stranded RNA, shRNA is a hairpin-structured RNA generated by transcription within cells via RNA polymerase III or II. This unique hairpin structure allows shRNA to stably exist within cells [98]. shRNA needs to be cleaved into siRNA before it can act on mRNA, so it can also be considered one of the sources of siRNA [99].

## 4. Co-Delivery of NPs-nc RNA by Nano-DDS

Despite the extraordinary potential of natural compounds and small RNAs, there are still many challenges. Firstly, the development of MDR in tumor cells limits the long-term therapeutic effects of NPs [100]. Secondly, the non-specific distribution of NPs in the body may cause toxic side effects on normal tissues during long-term administration, increasing the risk of treatment [101]. Moreover, most antitumor NPs are poorly water-soluble and toxic, so how to deliver them safely and effectively becomes a difficult problem [102]. The main component of the cell membrane is the phospholipid bilayer, which carries a negative charge, and small-RNA molecules also carry a negative charge [103]. Based on the mutual exclusion between the same charge, it is difficult for small RNAs to cross the cell membrane, affecting their function in the tumor cell. With free NPs or naked RNA administration into the vein, the drug half-life is not long, and they are easily cleared by the kidneys [104]. To overcome these challenges, researchers have employed nano-DDSs to encapsulate NPs and small RNAs, which not only effectively enhances their ability to penetrate biological membranes but also improves their targeting, reducing damage to normal cells and providing new ideas and methods for antitumor treatment.

The nano-DDS is an important vehicle in the current drug delivery field [22]. The nano-DDS consists of nanocarriers and drugs, where nanocarriers can include various types, such as liposomes, polymer nanoparticles, and inorganic nanoparticles, and drugs cover small-molecule drugs and nucleic acid drugs [105]. In this system, the efficient delivery of drugs is achieved by coating or adsorbing them onto the surface or inside of the nanocarrier, with the aid of the unique properties of the carrier. Nano-DDSs have various preparation processes, such as the self-assembly method, emulsification method, co-precipitation method, and layer assembly method. The most appropriate preparation process can be accurately selected according to the characteristics of different drugs and carriers [106]. In contrast, the pure drug-assembled nanosystem (PDNA), as a special derived form of the nano-DDS, is completely rid of its dependence on external carriers [107]. The PDNA is spontaneously aggregated by the drug molecules with the help of non-covalent interactions. The drug molecules play the dual roles of structural units and functional units, and they skillfully realize the self-delivery function of drugs [108]. The preparation process of the PDNA mainly relies on the intrinsic self-assembly potential of the drug molecules. Through the precise regulation of the solvent, temperature, pH, and other key conditions, we can stimulate the drug molecules to form nanostructures independently. This preparation method significantly simplifies the process without going through cumbersome carrier synthesis and drug loading, thereby greatly reducing the production cost and process complexity. More importantly, the PDNA does not need to use external carriers, which effectively avoids the toxicity and immunogenicity risks brought about by traditional carrier materials and significantly improves the safety and effectiveness of drugs [109]. However, the application range of the PDNA has some limitations compared with the traditional nano-DDS, which is mainly applicable to those drug molecules with self-assembly properties [110]. The optimization of the design and preparation process of these vectors can further improve their efficacy and safety in antitumor therapy, allowing for more treatment options for cancer patients.

### 4.1. Co-Delivery of NPs-siRNA

The development of the nano-DDS for the co-delivery of NPs and siRNA for antitumor treatment is more extensive compared to that for miRNA and shRNA. The targets for siRNA delivery commonly include STAT3, PD-L1, VEGF, Survivin, Bcl-2, and others. We summarized eight co-delivery nanocarriers constructed in the past five years in Table 1 and will elaborate on several remarkable research findings in the following text. Wang and colleagues [111] constructed a dextran-based lipid nanogel via self-assembly to co-deliver PTX and siRNA. By delivering siRNA targeting the MDR1 drug resistance gene, the sensitivity of DROV cells to paclitaxel was increased. The researchers also evaluated the in vivo biosafety of the formulation through H&E staining of the organs. The results showed that no significant pathological changes were observed in the organs after administration, indicating the biosafety of the formulation. Kim et al. [112] prepared chitosan-coated nanostructured lipid carriers (P-NLC-Chi-siRNA) for the co-delivery of paclitaxel and PD-L1 siRNA using the hot homogenization method. This formulation exhibited potent anticancer activity in mice bearing breast cancer-resistant (MCF-7) cells. Moreover, P-NLC-Chi-siRNA significantly reduced the PD-L1 mRNA expression both in vitro and in vivo, leading to enhanced CD4+ and CD8+ T-cell responses, thereby augmenting the synergistic antitumor effect in tumor xenograft models. By co-delivering two different but related RNAs with natural compounds, we can better compare the antitumor effects of this combined delivery method. CPT can specifically inhibit Topo I, thereby stimulating apoptosis. GPR78 and clusterin are key proteins against chemoresistance. Researchers [113] co-delivered CPT with GPR78 siRNA/CLU siRNA via liposomes to cancer stem cells to enhance the chemosensitivity of tumor cells. Fan et al. [69] constructed a co-incorporated polymeric hybrid nanoparticle (CSNP) to co-deliver Cur and siRNA CCAT1 through self-assembly. The CSNP was composed of two amphiphilic copolymers, polyethyleneimine-poly (d,l-lactide) (PEI-PDLLA) and 1,2-distearoyl-sn-glycero-3-phosphoethanolamine-N, as the carrier materials, and was self-assembled through the thin-film dispersion and electrostatic interaction methods. The CSNP-mediated co-delivery of Cur and siCCAT1 effectively silenced CCAT1 and achieved a synergistic effect, thereby increasing the Bcl-2-mediated apoptosis in HT-29 cells, inhibiting the EMT-mediated migration of HT-29 cells, and triggering significant antitumor efficacy in vivo through biocompatible combination therapy during the treatment process. Improving the TME is one of the important research directions for current cancer treatment, and glycolytic pathways are becoming important targets for regulating immunosuppression [114]. Silencing PD-L1 can inhibit the glycolysis of tumor cells and affect the TME, but balancing the glycolysis of tumor cells is more effective than simply inhibiting glycolysis [115]. Researchers [116] developed a dual-responsive polymer complex (DRP/Res/siP) for the co-delivery of PD-L1 siRNA and Res to achieve antitumor effects by silencing PD-L1 to balance the glycolysis and mitochondrial oxidative phosphorylation of tumor cells. The studies show that in B16F10 and CT26 tumor mouse models, the DRP/Res/siP treatment group showed significant tumor inhibition effects, with tumor inhibition rates of 74.9% and 67.3%, respectively. DRP/Res/siP significantly increased the infiltration of CD8+ T cells and CD4+ T cells in the tumor and raised the secretion level of IFN-γ. Meanwhile, the DRP/Res/siP treatment significantly reduced the proportion of regulatory T cells (Tregs) and myeloid-derived suppressor cells (MDSCs), improving the immunosuppressive state of the TME.

### 4.2. Co-Delivery of NPs-miRNA

miRNA can target different sites in tumor cells to regulate gene expression [121]. Recent studies on the construction and evaluation of nanocarriers for the co-delivery of NPs-miRNA are summarized in Table 2. Wang et al. [122] constructed multivalent, rubber-like RNA nanoparticles for the co-delivery of paclitaxel and miR122, enhancing the therapeutic effect on liver cancer through the passive EPR effect and active tumor targeting with tumor-specific ligands. ADAM10 is a membrane-bound metalloprotease belonging to the ADAM family [123]. Efficient delivery enabled miR122 to effectively silence ADAM10 mRNA, and miR122 could inhibit the expression of drug efflux transporters, thereby overcoming hepatocellular carcinoma (HCC)’s resistance to PTX and sensitizing HepG2 cells to PTX treatment, and thus synergize with PTX. Zhou et al. [124] constructed nanoparticles for the co-delivery of paclitaxel and miR221/222, which could simultaneously deliver PTX and miR221 or miR222 for the treatment of TNBC. This formulation could upregulate the expression of p27^kip1^ and TIMP3 in MDA-MB-231 cells, achieving a tumor-inhibiting effect. The study results indicated that when paclitaxel was co-delivered with miR221 and miR222 via nanoparticles, the cell viability of the MDA-MB-231 cells was significantly reduced. The nano-DDS is usually modified with certain ligands to enhance its targeting effect and achieve more precise delivery [125]. Researchers [126] developed lactobionic acid (LA)-modified lipid nanoparticles (LNPs) for the co-delivery of camptothecin and miR-145 for the treatment of HCC. The LA modification improved the targeted delivery of drugs to HCC cells and tissues. miR-145 may target SENP1-mediated HK2, disrupt their binding, and induce apoptosis through the glycolysis pathway, thereby sensitizing cancer cells to CPT. And the co-delivery of CPT and miR-145 was more effective than the delivery of CPT or miR-145 alone and formulation development involving the co-delivery of Cur, RES, and miRNA. Additionally, this study monitored the body weight and liver function indices in serum after administration. The results indicated that, compared with the model group, LA-CMGL could reduce the levels of ALT and AST in serum, demonstrating the formulation’s good biosafety. Li et al. [127] constructed ROS/GSH dual-sensitive nanoparticles for the co-delivery of curcumin and miR155 (CUR/miR155@DssD-Hb NPs) for the synergistic immunotherapy of breast cancer. The targets of miRNA are not necessarily specific target genes but may also affect cell immunity through complex multiple actions [128]. The elevated levels of miR155 in breast cancer cells have been shown to inhibit tumor progression by increasing the recruitment of antitumor immune cells. Curcumin, due to its potential in cancer immunotherapy, has become a good partner for co-delivery with miR155. The construction of conventional nanocarriers still has certain limitations in delivery. Researchers encapsulated Cur in the cores of nanoparticles through self-assembly polymerization, while miR155 was loaded onto the surfaces of nanoparticles through electrostatic adsorption. When CUR/miR155@DssD-Hb NPs encounter the high levels of ROS commonly present in cancer cells, the gradual “self-destruction” mechanism, which involves the transformation of hydrophobic groups (boronate) to hydrophilic groups (carboxyl), helps to effectively release CUR and miR155.

### 4.3. Co-Delivery of NPs-shRNA

The properties of shRNA are similar to those of siRNA. Currently, the development of the nano-DDS for the co-delivery of NPs and shRNA is still limited. This may be because shRNA can be cleaved by Dicer enzymes to form siRNA, serving as one of the sources of siRNA [130]. Moreover, shRNA needs to be cleaved into siRNA to exert its function within cells, and its efficiency is lower than that of siRNA and miRNA [99]. However, with the continuous development of material technology, more nano-DDSs are available to achieve the more efficient delivery of shRNA. Survivin is a common target for shRNA. Hu et al. [131] co-delivered Survivin shRNA and PTX via supramolecular micelles for the synergistic treatment of ovarian cancer. Additionally, Babaei et al. [132] constructed rod-shaped mesoporous silica nanoparticles, and Sanati et al. [133] developed anionic dextran-coated micelles for the co-delivery of CPT and Survivin shRNA for the treatment of colon cancer. The targeting of nano-DDSs is generally passive targeting [134], and appropriate ligand modification during the construction of nanocarriers can enhance their active targeting [135]. Folic acid is a widely used active targeting ligand [136]. Jia et al. [137] constructed the co-delivery of PTX and P-shRNA by FA-mediated silica nanohybrids for reversing MDRA.

### 4.4. Nanocarriers Can Enhance the Targeting of Drug Co-Delivery

Nanocarriers can co-deliver NPs and small RNAs to specific target sites, mainly by optimizing the preparation design of the nanocarriers. Due to the EPR effect, nanocarriers inherently possess passive targeting capabilities. However, to enhance the active targeting ability of nanocarriers, targeting ligands that can be modified on the surface are often added. For example, folic acid, which is highly expressed in tumors but minimally in normal cells, is frequently used to modify nanocarriers to enhance their tumor-targeting abilities [138]. Alternatively, cell membranes can be used to coat nanocarriers to enhance the active targeting. For instance, tumor cell membranes can encapsulate nanocarriers using a “self-recognition” mechanism to aggregate the delivery system at the tumor site and evade immune clearance [139]. After identifying the target tumor for nanocarrier delivery, the corresponding target site for that tumor can be selected to modify the nanocarrier. This method achieves the co-delivery of NPs and small RNAs to target cells or tissues.

## 5. Concluding Remarks

This study focuses on the research progress in the co-delivery of natural active ingredients and small RNAs, both of which are key drugs currently used for antitumor treatment. As of the research up to 2025, there are still countless studies proving that natural compounds have a close relationship with the related RNA expression in the antitumor process. Delivering relevant small RNAs to target genes via nanocarriers and combining it with the delivery of natural compounds can form a synergistic effect within tumor cells, effectively inhibiting tumor growth. The particle size of the resulting nanocarrier usually is slightly larger due to the co-delivery of the double-carrier drug and the addition of the distribution medium [140]. But generally speaking, keeping the particle size below 200 nm is enough to achieve a good delivery effect. Nanocarriers as delivery vehicles for small RNAs can help enhance the stability of small RNAs, but we still need to employ some methods to optimize the stability of the delivery. For example, mixing protamine with siRNA to form a complex first and then incubating this complex with nanocarriers can result in a more stable small-RNA delivery system [141]. It should also be noted that endogenous Argonaute proteins play a crucial role when small RNAs form complexes with the RISC. These proteins are widely present in cells and can bind with small RNAs to create the RISC, which then mediates gene silencing [142]. If the expression level of endogenous Argonaute proteins is low or their function is inhibited, exogenous supplementation of Argonaute proteins may be required to enhance the efficacy of small-RNA drugs. Future research could delve deeper into the role of endogenous Argonaute proteins in antitumor effects, which would aid in the optimization of nanocarrier designs for co-delivery.

Although the co-delivery of small RNAs and NPs in nano-DDSs has made some progress in the current research, there are still challenges, such as the complex preparation processes, high costs, small sample sizes in clinical trials, short follow-up periods, and impact of individual differences on treatment outcomes. So far, few co-delivery formulations of NPs and small RNAs have achieved remarkable results in clinical studies. This indicates that the research achievements for such formulations are still at the laboratory stage. Future research can further optimize the design and preparation of nanocarriers to improve their targeting, stability, and biocompatibility, and explore more co-delivery antitumor mechanisms of NPs and small RNAs, for example, by adding different targeting heads or ligand modifications to enhance the targeting of nano-DDSs, or by combining them with other means, such as photothermal therapy, to improve the therapeutic effect of co-delivery formulations. At the same time, deeper research on the drug resistance mechanisms of tumor cells and the specific effects of small RNAs in tumors will help develop more precise and effective antitumor strategies, fully utilize the potential of NPs and small RNAs, bring about better treatment options for cancer patients, and achieve more efficient, precise, and safe cancer treatment. We also suggest that when researchers design and construct co-delivery systems for nanocarriers, they should consider conducting in vitro or in vivo safety evaluations of the developed products. For example, in vitro hemolysis tests can be performed for intravenous formulations, and comprehensive in vivo biosafety assessments can be conducted through the monitoring of animal body weights, the microscopic observation of organ structures, and the evaluation of liver and kidney function indicators in serum. In summary, the research findings compiled in this review provide new ideas and directions for the development of future antitumor drugs. We have reason to believe that in the future, this co-delivery design concept will play an increasingly important role in the field of tumor treatment and bring new hope and light for humanity to overcome cancer.

## Figures and Tables

**Table 1:** Co-delivery of NPs and siRNA in antitumor nano-DDS.

NP	Target Spot	Cancer	Characterization			Reference
			Size (nm)	Zeta (mV)	EE (%)	
PTX	MDR1	Ovarian cancer	100	-	93 87.6 (RNA)	[111]
PTX	PD-L1	Breast cancer	181.97	18.66	99.96	[112]
CPT	GRP78 clusterin	Breast cancer	281.7 (CLU) and 242.7 (GRP78)	48.89 (CLU) and 52.84 (GRP78)	-	[113]
CPT	PLK-1	Cervical cancer and breast cancer	60	-	-	[117]
Cur	STAT3	Skin cancer	195.0	58.8	88.9	[118]
Cur	Bcl2	Cervical cancer	160–180	-	-	[119]
Cur	CCAT1	Colon cancer	180	−10.48	97	[120]
RES	PD-L1	TME	118.2	~10	8.80 (RNA)	[116]

**Table 2:** Co-delivery of NPs and miRNA in antitumor nano-DDS.

NP	Target Spot	Cancer	Characterization			Reference
			Size (nm)	Zeta (mV)	EE (%)	
PTX	ADAM10	Liver cancer	17.3	-	-	[122]
PTX	p27^kip1^ and TIMP3	Triple-negative breast cancer	100	-	-	[124]
CPT	HK2 and VDAC1	Liver cancer	160–170	−3.5	85 (CPT) 81 (RNA)	[126]
Cur	Immunocyte	Breast cancer	121.56	15.35	98.36 (RNA)	[127]
RES	Apoptosis-related gene	Osteosarcoma	257	29.2	~85 (RNA)	[129]

## Data Availability

No new data were created or analyzed in this study. Data sharing is not applicable to this article.

## Abbreviations

The following abbreviations are used in this manuscript:

CPT	Camptothecin
Cur	Curcumin
Dicer	Double-stranded RNA-specific ribonuclease
dsRNA	Double-stranded RNA
EPR	Enhanced permeability and retention
MDR	Multidrug resistance
miRNA	MicroRNA
PTX	Paclitaxel
Nano-DDS	Nanomaterial-based drug delivery system
NPs	Natural products
PDNA	Pure drug-assembled nanosystem
RES	Resveratrol
RISC	RNA-induced silencing complex
shRNA	Short hairpin RNA
siRNA	Small interfering RNA
RNAi	RNA interference
ROS	Reactive oxygen species
TME	Tumor microenvironment
TNBC	Triple-negative breast cancer
